# Polyethylene glycol triggers the anti-cancer impact of curcumin nanoparticles in sw-1736 thyroid cancer cells

**DOI:** 10.1007/s10856-021-06593-9

**Published:** 2021-08-28

**Authors:** Simzar Hosseinzadeh, Hojjatollah Nazari, Elaheh Esmaeili, Shadie Hatamie

**Affiliations:** 1 Medical nanotechnology and tissue engineering research center, Shahid Beheshti University of Medical Sciences, Tehran, Iran; 2 Department of Tissue engineering and Applied Cell Sciences, School of Advanced Technologies in Medicine, Shahid Beheshti University of Medical Sciences, Tehran, Iran; 3 Department of Medical Nanotechnology, School of Advanced Technologies in Medicine, Iran University of Medical Sciences, Tehran, Iran; 4 Stem cell technology research center, Tehran, Iran; 5 Institute of NanoEngineering and MicroSystems National Tsing Hua University Hsinchu, 30013 Hsinchu, Taiwan, ROC; 6 Department of Power Mechanical Engineering National Tsing Hua University Hsinchu, 30013 Hsinchu, Taiwan, ROC

## Abstract

Curcumin has been recognized as an effective anticancer agent. However, due to its hydrophobic property, the cell absorption is not satisfied. Herein, the curcumin nanoparticles were prepared in the presence of polyethylene glycol 6000 (PEG6000) to reduce its elimination by immune system. For first time, not only the curcumin was encapsulated within the niosome nanoparticles modified by PEG, there are no reports related to the anticancer property of curcumin against thyroid cancers. The nanoparticles was developed and its anticancer was studied on sw-1736 cancer cell line. The nanoparticles were examined by scanning electron microscopy (SEM) and dynamic light scattering (DLS). Also, the release profile of curcumin, the IC50 concentration, the radical amount and the gene expression were evaluated. The optimized nanoparticles showed a diameter of 212 ± 31 nm by SEM and the encapsulation efficiency and loading capacity of 76% and 16.8% respectively. DLS confirmed the polydispersity index (PDI) of 0.596 and the release model was shown a sustained release with the delivery of 68% curcumin after 6 days. Also, the nanoparticles indicated the higher storage stability at 4 °C. After the cell treatment, the apoptotic bodies were appeared and IC50 was obtained as 0.159 mM. Moreover, the generated radicals by the treated cells was 86% after 72 h and the gene pattern indicated the bax/bcl2 ratio of 6.83 confirming the apoptosis effect of the nanoparticles. The results approved the nanoparticles could be suggested as an anticancer drug candidate for thyroid cancers.

**Figure:**
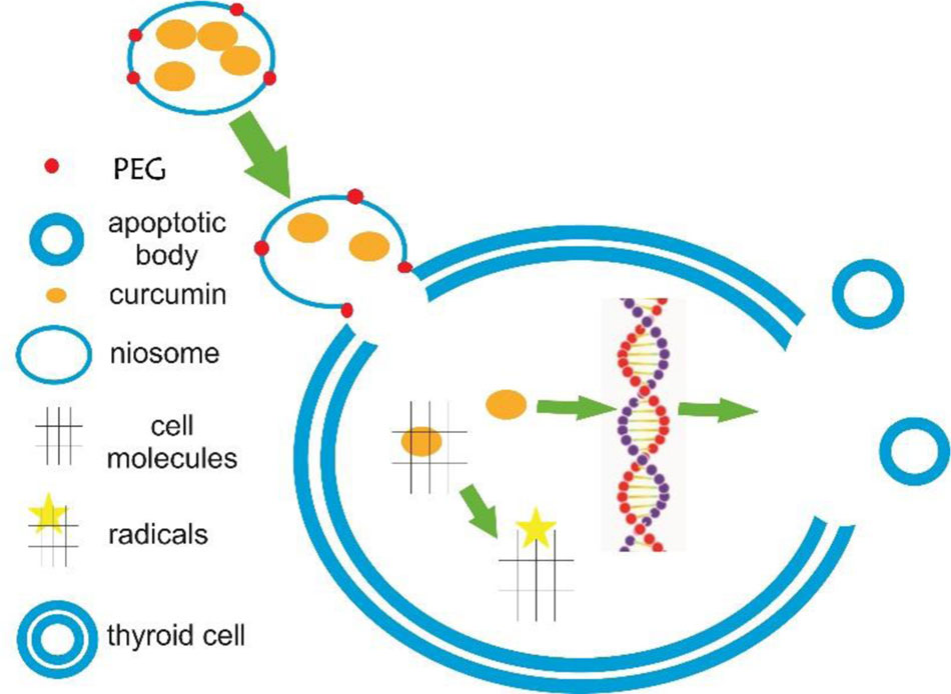
The encapsulated curcumin within the niosome nanoparticles modified with PEG, could be released and up-taken by the thyroid cancer cell line due to the same hydrophobic property of cell membrane and the niosome particles. The reaction between curcumin and cellular components generates radicals and activates the apoptotic pathway. The corresponding reaction finally makes cell death.

The encapsulated curcumin within the niosome nanoparticles modified with PEG, could be released and up-taken by the thyroid cancer cell line due to the same hydrophobic property of cell membrane and the niosome particles. The reaction between curcumin and cellular components generates radicals and activates the apoptotic pathway. The corresponding reaction finally makes cell death.

## Introduction

Cancer has been considered as one of the main causes of death in all over the world and it is defined as an uncontrolled proliferation and expansion of abnormal cells [1, 2]. In accordance with before reports, cancers may appear in different organs and it has a wide range of origins including including DNA mutation [3], viruses [4], or the environmental carcinogenic agents [5]. Now, both developed and developing countries are facing with the increasing rate of cancer incidence [6]. Thyroid cancers are known among the most common malignancies in the endocrine system neoplasm [7]. These types of cancers can originate from the follicular or the parafollicular thyroid cells. The follicular thyroid cells are the source of follicular thyroid carcinoma, poorly differentiated thyroid carcinoma, papillary thyroid carcinoma (PTC), and anaplastic thyroid carcinoma (ATC). On the other hand, the medullary thyroid cancer is derived from parafollicular cells. However, about 75–85% of thyroid cancer cases are involved with PTC [8, 9]. Also, the development of cancer cells could be categorized into the well-differentiated and poorly differentiated types and almost 95% of thyroid cancers are belonged to these differentiated cells [10]. Surgical therapies, chemotherapy method, radioactive iodine therapies, hormone therapy, radiation therapy, and targeted therapies are prevalent therapeutic methods for the treatment of thyroid cancer in clinic [11, 12]. In new challenges to accumulate an anticancer drug in thyroid cancer cells, a group used pH-responsive nanoparticles [13]. Other group employed nanoclay particles for the delivery of doxorubicin to papillary thyroid cancer cells and the results approved a controlled diffusion rate [14]. A similar study benefitted nanobubbles for the doxorubicin delivery to anaplastic thyroid cancer cells [15]. Also, methacrylated glycol chitosan polymer was recruited for the synthesis of the nanoparticles loaded doxorubicin [16]. However, the low response to the high toxic result of chemotherapeutic drugs, the dosimetric limitations in iodine therapy, potential side effects, drug resistance issue and also, the recurrence of cancer are unsolved challenges about cancer therapy approaches that need to be addressed. In spite of chemical drugs, some folk drugs such as curcumin, are introduced as a folk medicine for cancer therapy. However, the final effect of these folk drugs on cancer therapy is not so impressive. Advanced sciences and technologies including nanotechnology play an important role in this area. Curcumin is using as a traditional folk drug for the treatment of different cancers. Curcumin (1,7-bis(4-hydroxy-3-methoxyphenyl)-1,6-heptadien-3,5-dione) is a lipophilic molecule with a high permeability through cell membrane [17]. This material is considered as an active ingredient in turmeric [18]. A variety of cancer studies have been performed about curcumin due to its innate properties including; antioxidant activity, anti-angiogenesis, antitumor and anti-inflammatory properties. Also curcumin inhibits cell proliferation and induces apoptosis in different cancer cells including leukemia, pancreatic [19], prostate [20], colorectal [21], and lung [22]. In cancer molecular studies, it has been confirmed that curcumin suppresses several signaling pathways, leading to a significant decrease in cell proliferation, metastasis, and the angiogenesis abilities of cancer cells. Some of these activated pathways, directly or indirectly induce apoptosis and the death of cancer cells. Curcumin binds to different proteins inside cancer cells, including 5-lipoxygenase (5-LOX), thioredoxin reductase, cyclooxygenase-2, (COX2), protein kinase C, and tubulin [23]. Curcumin also can induce apoptosis in cancer cells via the variety of apoptotic and growth inhibitory pathways. The majority of these pathways release apoptosis factors to activate caspase [24], and regulate the cell cycle [25]. Also, these factors inhibit the signaling profile of COX2 and 5-LOX [26, 27] and activate pro-oxidant/anti-oxidant mechanisms [28] and mitochondrial [24, 29, 30] pathways. Curcumin induces apoptosis in cancer cells not in normal cells [31]. For instance, this material also effects on cell cycle regulation and induces apoptosis at G2 phase in mammary epithelial carcinoma cells and usually leave normal cells healthy [32]. This lower sensitivity of normal cells to curcumin has been occurred due to their lower metabolism rate compared to cancer cells. Despite the all mentioned properties of curcumin for cancer therapy, it cannot be accepted as a high efficient anticancer drug because of its high hydrophobic nature. Nowadays, nanotechnology-based pharmaceutics have introduced the different types of nanoparticles as nanocarriers to enhance the effectiveness of anticancer drugs such as curcumin [33]. A various range of drugs have been loaded into liposomes, polymeric nanoparticles, dendrimers, and niosomes for targeted drug delivery [34, 35]. The low efficiency of the curcumin delivery is related to its poor bioavailability. Also, the positive effect of curcumin is limited due to its fast metabolism and rapid systemic elimination. There are some nanocarriers that enhance and improve the therapeutic effects of curcumin by increasing its bioavailability and solubility. Also, they could protect it from enzymatic degradation reactions. These nanocarriers also provide a controlled release for curcumin and can limit their nonspecific uptake. Bisht et al. (2007) encapsulated curcumin into nanomicelles with the diameter of 50 nm made of N-isopropylacrylamide, N-vinyl-2-pyrrolidone and poly (ethyleneglycol) monoacrylate. The nanoparticles showed an acceptable therapeutic result in comparison to the free curcumin against a human pancreatic cancer cell lines via apoptosis induction process [36]. Das et al. (2010) incorporated curcumin into composite biocompatible nanoparticles made of alginate, chitosan and pluronic, and investigated their cellular internalization [37]. Also, Duan et al. (2010) loaded curcumin into chitosan/poly (butyl cyanoacrylate) nanoparticles and investigated its effect in the in vitro and in vivo studies. These nanoparticles inhibited hepatocellular carcinoma growth and angiogenesis in murine xenograft models [38]. Mulik et al. (2010) entrapped curcumin into the transferrin-mediated solid lipid nanoparticles. Their studies revealed that the uptake and apoptotic anticancer activity of curcumin is enhanced after its loading into the nanoparticles [39]. Zhang et al. (2017) developed pH-sensitive polymeric nanoparticles as carriers for doxorubicin and curcumin. These nanoparticles enhance releasing activity, cellular internalization and apoptosis rate in the SMMC 7721 cells through the reduction of the mitochondrial membrane potential [40]. Kalashnikova et al. (2017) loaded curcumin into dextran coated nanoparticles. They found that these nanoparticles induce a substantial cell death in neuroblastoma cells via prolonged oxidative stress and ROS-mediated apoptosis by the modulation of Bcl2/Bax [41]. Niosomes are the nanocarriers formed by the self-association of nonionic surfactants and cholesterol in an aqueous phase [42] that attract attentions for the delivering of a variety of drugs. These nanoparticles have been used for the curcumin delivery to the lung cancer cells. Jyoti et al. (2016) synthesized the inhalable cationic niosome loaded curcumin to overcome physicochemical and biopharmaceutical barriers against the lung cancer cells. The prepared niosomes increased apoptosis, intracellular uptake, internalization and cytotoxicity in A549 non-small cell lung cancer cells [43]. However, for higher circulation half-life and lower phagocytosis of niosomes, niosomes must be coated with a biocompatible and hydrophilic polymer. In a study, curcumin and paclitaxel were co-loaded in niosomes which were coated by polyethylene glycol (PEG) for the application of breast cancer therapy [44]. Again, another study employed PEG as a stabilizer for the preparation of niosome nanoparticles loaded by quercetin [45]. Moreover, PEG could increase the tumor targeting of niosomes which were used for imaging approaches [46, 47]. Beside the benefits of curcumin formulation, it would be better that some drawbacks are noted. One of the main disadvantage of niosomes is associated to their low reproducibility due to a changeable purity of phospholipid [48]. Moreover, their production is expensive and difficult [49] and also, they are unstable at different pH levels [50]. On the other hand, as above mentioned, curcumin has poor water solubility reducing its bioavailability [51]. In addition, it was confirmed that phytochemicals as curcumin have a lower stability in biological environments [52] due to their high metabolism rate [51]. In spite of this, the formulation of curcumin in the presence of PEG enhances its solubility. However, PEGylation decreases the cellular absorption of nanoparticles and therefore, the low activity of that formulation is inevitable [53].

Herein, for the first time, the niosome based nanoparticles was used as the carriers for curcumin delivery to thyroid (sw-1736) cancer cell line. In accordance with a complete literature review, there is no reports related to the application of anticancer niosomes against thyroid cancer cells. Also, as a pioneer study, the nanoparticles were modified by PEG leading to higher circulation time in body after intravenous administration. The prepared nanoparticles were studied to obtain its kinetic release rate of curcumin and biological assays including cell treatments, created radical amount and also, gene expression pattern and the nanoparticles stability were analyzed in the following.

## Materials and methods

### Materials

Curcumin (purity > 99%,), Tween-80, span 80, cholesterol and PEG 6000 (PEG6000) were purchased from Sigma-Aldrich (St. Louis, MO, USA). Ethanol (≥99.5%) which was used as a solvent and dimethyl sulfoxide (DMSO) for cell culture were obtained from Merck (Shuchardt, Germany). Cell culture materials including low glucose Dulbecco’s Modified Eagle Medium (DMEM), fetal bovine serum (FBS), trypsin-EDTA, penicillin, streptomycin, phosphate buffer solution (PBS) and MTT (3-[4, 5-dimethylthiazol-2-yl]-2, 5-diphenyl tetrazolium bromide) were bought from Gibco (AG, basel, Switzerland). Also, 1,1-diphenyl-2-picrylhydrazyl (DPPH) was provided from Sigma-Aldrich (St. Louis, MO, USA). A dialysis bag for release measurements with the molecular mass cut-off between 12 kDa and 14 kDa, was purchased from Sigma-Aldrich (St. Louis, MO, USA).

### Preparation of noisome encapsulated curcumin nanoparticles

A solution was prepared by using Tween-100 (50 µl/ml), span 80 (15 µl/ml) and cholesterol (15 µl/ml) in the ethanol solution of PEG6000 (10%). After the achievement of a uniform solution, the substrate (curcumin) was added to the solution at the concentration of 3 mM. The final mixture was vortexed till the solution to be cleared. Finally, the complex of curcumin with the surfactants was added drop wise to the heated distilled water at 80 °C and mixed by continuous stirring at the ratio of 1:50 (solution: water, v/v). Then, the solution temperature was decreased to 45 °C for 30 min while continuous stirring. In this stage, by using the hydrating process, the noisome nanoparticles are getting form. The obtained particles were washed for several times to clean the particles from the non-conjugated PEG. For the non-capsulated curcumin removal, the final 50 ml of the niosome solution in distilled water was dialyzed using a polymer dialysis bag (molecular mass cut-off between 12 kDa and 14 kDa) against 100 ml of deionized water for 24 h. During this step, the free curcumin was removed out and the niosome sample was remained in the bag. The solution of the dialysis bag was centrifuged at 22000 *rpm* with the temperature of 4 °C and then dispersed in 1 ml of distilled water. Afterwards, the final suspension which was ultra-sonicated for 20 min, was used for the following assays.

### Particle size of niosome encapsulated curcumin nanoparticles

The morphology of the noisome *encapsulated* curcumin was examined by scanning electron microscopy (SEM; Philips, XL-30) after gold sputtering. The applied magnification was ×30,000 to see small particles near nanometer. The particles were studied by SEM after washing, dialysis, ultrasonic and dilution steps. The size distribution of the diluted nanoparticles (particle dispersion index (PDI)) and their average particle size were studied by dynamic light scattering (DLS) (Malvern Instruments, Worcestershire, UK) at room temperature. The assay was repeated for three times and also, after the dilution of the suspension to ten times, the assay was repeated.

### Encapsulation efficiency of niosome encapsulated curcumin nanoparticles

A standard curve was required to calculate the encapsulation efficiency of curcumin in the niosome particles. The curve was drawn by using a gradient concentration of the free curcumin from 0.036 to 3 mM. The absorbance of the curcumin solution samples was read at 425 nm. After the removal of the free curcumin by using the dialysis bag, the optical density (OD) of the filtrate (solution filtered out) was read at 425 nm and the obtained value of OD was changed to a curcumin amount (g) by using a standard curve. In accordance with the below formula, the encapsulation efficiency and loading capacity were calculated:

Encapsulation efficiency (%) = [initial curcumin (g) − free curcumin (g)]/initial curcumin (g) × 100

Loading capacity (%) = [initial curcumin (g) − free curcumin (g)]/total nanoparticle (g) × 100

### Curcumin release from niosome encapsulated curcumin nanoparticles

The percentage of the curcumin release was measured against time in accordance with the values of the standard curve. For the release measurement, the resultant solution was incubated for 72 h at 37 °C in PBS and at the times of 2, 4, 8, 24, 48 and 72 h, 1 ml was withdrawn for the UV-Vis spectrophotometry at the λ_max_ of 425 nm and substituted with 1 ml of the fresh PBS. The test was repeated and the average number with the standard deviation (SD) was calculated. The concentrations of the released curcumin, were obtained after the adjustment of the OD values with the standard curve. The kinetic of the released curcumin was evaluated using Peppas equation according with the following formula [54, 55]:

$${{{{{\mathrm{M}}}}}}_{{{{{\mathrm{t}}}}}}/{{{{{\mathrm{M}}}}}}_\infty = {{{{{\mathrm{K}}}}}}_{{{{{\mathrm{m}}}}}}{{{{{\mathrm{t}}}}}}^{{{{{\mathrm{n}}}}}}$$

Herein, M_t_ is the accumulative amount of the released curcumin at the time t, M _∞_ presents the total amount of the encapsulated curcumin which had been calculated via the encapsulation efficiency formula in the previous section. Also, the free curcumin was dissolved in DMSO at the concentration of the encapsulated amount in the niosome nanoparticles and the related OD absorbance values were read at the above mentioned time points. The assay was done same for the free curcumin and at each time point, 1 ml of the curcumin solution was replaced with the fresh PBS and the absorbance was read at 425 nm.

### Stability of niosome encapsulated curcumin nanoparticles

The stability of the prepared niosome particles was evaluated by the measurement of size change rate (SCR) and also by the loading leakage. The all samples were kept in the tightly closed colored containers at 4 and 37 °C. The storing times were 0, 15 and 30 days for the examination of particle size and 0, 12, 18, 24 and 30 days for the measurement of the curcumin leakage. The size changes and the retained curcumin were calculated by SEM and the absorbance at 425 nm respectively. The SCR values were calculated in accordance with the below formula:

$${{{{{\mathrm{SCR}}}}}} = \left( {{{{{{\mathrm{St}}}}}}-{{{{{\mathrm{S0}}}}}}} \right)/{{{{{\mathrm{S0}}}}}} \times {{{{{\mathrm{100}}}}}}$$

Where St determines the size at a time point and S0 is the initial size. Also, for the measurement of curcumin that was leached out, a 0.5 ml of the niosome suspensions was withdrawn and centrifuged, then the absorbance was read at 425 nm. The process was repeated for the all time points.

### Cell viability after the treatment with the niosome encapsulated curcumin nanoparticles

Sw-1736 human thyroid cancer cell line and Hu02 human foreskin fibroblast cell line as the cancer and normal cell lines respectively, were purchased from stem cell technology research center Tehran, Iran (code: BN-0012.1.26). The cells were counted and seeded in 96 well plates at 10 × 10^3^ per well and incubated with DMEM-10% FBS at 37 °C in a 5% CO2 humidified atmosphere. After 24 h, the cell groups were divided as the control and test groups. The test groups were treated with a serial dilution of the niosome nanoparticles obtained after washing, dialysis and ultra-sonication (batch sample). The concentrations of the nanoparticles for this serial dilution were 0, 0.5, 2, 7, 12 and 15 µl of the batch sample in 100 µl of cell culture media. The alive cell numbers were studied via the MTT process as the following after 24, 48 and 72 h. For this assay, at a predetermined time, the wells were precisely washed with PBS and incubated with MTT (0.1 mg/ml in DMEM without FBS). After 3.5 h, the MTT solutions were aspirated and then, DMSO was added to solve the formazan crystals and at last, their absorbance was read at 570 nm. For the calculation of cell viability percentages, the OD values of the treated groups were normalized against TCPS in accordance with the below formula:

$${{{{{\mathrm{Cell}}}}}}\,{{{{{\mathrm{viability}}}}}}\% = \left( {{{{{{\mathrm{OD}}}}}}_{{{{{\mathrm{t}}}}}}-{{{{{\mathrm{OD}}}}}}_{{{{{\mathrm{c}}}}}}} \right)/{{{{{\mathrm{OD}}}}}}_{{{{{\mathrm{c}}}}}}{{{{{\mathrm{100}}}}}}$$

Where, OD_t_ and OD_c_ are the representative of absorbance values related to the treated and non-treated groups respectively.

### Analysis of apoptotic genes after cell treatment

TRIzol reagent (Sigma-Aldrich, St. Louis, MO, USA) was used to isolate the total RNA of the treated cells. cDNA was synthesized with the M-MuLV reverse transcriptase (RT) and Random Hexamer primers, according to the manufacturer’s instructions (Fermentas, Life science, Canada). The PCR reactions (94° for 3 min as the annealing temperature, 35 cycles as 94° for 30 s, 62° for 45 s, 72° for 45 s and the extension time was 72° for 7–10 min) were conducted with 0.5 μl of the cDNA product. The Real-Time PCR reactions were performed using MaximaTM SYBR Green/ROX Real-Time PCR Master Mix (Fermentas, Life science, Canada) and Rotor-gene Q software (RG- 6000, Corbett Research, CA, USA) for the data analysis of threshold cycle average. The gene expression levels were calculated based on the ∆∆Ct method. For this assay, the Ct numbers of the specific genes were normalized with beta2 microglobolin (beta2M) as the internal control and the obtained values were calibrated against TCPS. The difference between test group and TCPS was reported as significant relations when *p* value was ≤0.05. The primer sequences were listed in Table 1.

**Table 1:** The primer sequences of the studied genes related to the apoptosis process

Name	Host	Sequence
Beta2M - F	Human	ATG CCT GCC GTG TGA AC
Beta2M - R	Human	ATC TTC AAA CCT CCA TGA TG
BCL2-F	Human	GTA CTT AAA AAA TAC AAC ATC ACA G
BCL2-R	Human	CTT GAT TCT GGT GTT TCC C
BAX-F	Human	CAA ACT GGT GCT CAA GGC
BAX-R	Human	CAC AAA GAT GGT CAC GGT C
Caspase3-F	Human	GTGGAACTGACGATGATATGGC
Caspase3-R	Human	CGCAAAGTGACTGGATGAACC
Caspase8-F	Human	CCG AGC TGG ACT TGT GACC
Caspase8-R	Human	CTG CCC AGT TCT TCA GCA AT

### Measurement of free radical amount by DPPH assay

The free radical values were quantified in the absence and presence of the treated sw-1736 thyroid cancer cell line by the niosome encapsulated curcumin nanoparticles. Then, the determined groups were incubated by a solution of DPPH diluted in methanol to reach the final concentration of 1 mM. The samples were kept in the darkness and the absorbance values at 520 nm were obtained after 30 min [56] to measure the ROS amount generated by the treated cells or curcumin itself.

### Statistical analysis

The statistical analysis was done by using sigma-plot software (version 10, Systat Software Inc., Richmond, CA, USA) for the assays. The student’s *t* test was used to evaluate the differences between the data means of the experimental and control groups. The *p* values ≤ 0.05 was taken statistically significant and the related data were presented as mean ± SD. For the calculation of the significant relations between the groups in Real-Time PCR, the Ct numbers related to the specific genes were normalized with beta2M as the internal control and the obtained values were calibrated against TCPS. The difference between the test group and TCPS was reported as a significant relation. About DPPH assay, the all groups (test groups) were compared to the group contained only PBS (control group). For the achievement of IC50 value, MTT characterization was employed and the values of the different concentrations were compared to the group without any nanoparticles (control group). If the obtained value of the test groups was about 50% of the OD related to the control group, the corresponding nanoparticle concentration was reported as IC50.

## Results

### Encapsulation efficiency and loading capacity of curcumin in niosomes nanoparticles

In accordance with Table 2, the different synthesis conditions developed the various encapsulation efficacies. After the dialysis, the encapsulation value was obtained via the spectroscopic absorbance of the free curcumin washed out from the dialysis bag at 425 nm. The concentration of curcumin in the all synthesis groups were same and equals to 3 mM, but the entrapment percent was different between the various formulations. Herein, the encapsulation efficiency was increased with the groups contained PEG compared to the formulations without this polymer. Also, a new composition was developed by using PEG and cholesterol simultaneously to get their synergic effect on the efficiency. The results confirmed the highest curcumin encapsulation efficiency as 76% compared to the other synthesis conditions. Also, the values of the loading capacity were calculated for the all synthesis groups. Herein, the mass of the resultant nanoparticles was 0.05 gr. The data confirmed that after the addition of cholesterol, the loading capacity was increased from 5.8 to 9.6% and even, PEG could not enhance the value in the absent of cholesterol (formulation number 4 with the loading capacity of 7.4%). However, similar to the encapsulation efficiency, PEG and cholesterol had a synergic influence on the higher loading capacity as 16.8%.

**Table 2:** The encapsulation efficiency and loading capacity of the different formulations in this study

Groups	Components	Initial cur. (M)	Initial cur. (g)	Free cur. (M)	Free cur. (g)	Encapsulation efficiency (%)	Loading capacity (%)
1	Tween/span/cur.	3 × 10^−3^	0.011	22 × 10^−4^	0.0081	26	5.8
2	Tween/span/cholesterol/cur.	3 × 10^−3^	0.011	17 × 10^−4^	0.0062	43	9.6
3	Tween/span/cholesterol/PEG/ cur.	3 × 10^−3^	0.011	72 × 10^−5^	0.0026	76	16.8
4	Tween/span/PEG/cur.	3 × 10^−3^	0.011	20 × 10^−4^	0.0073	33	7.4

### Particle size of curcumin niosome nanoparticles via SEM and DLS

**Fig. 1:**
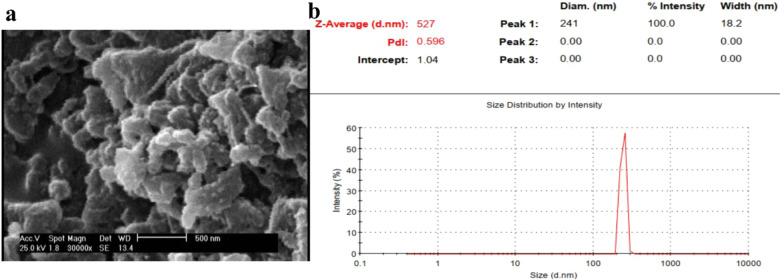
SEM morphology by the magnification of ×30,000 approves the diameter of the nanoparticles as 212 ± 31 nm (**a**). DLS study of the curcumin niosome nanoparticles after washing, ultrasonic sonication and dilution steps reports the resultant diameter as 241 nm with PDI of 0.596

Figure 1a shows the SEM morphology of the prepared curcumin niosome particles in accordance with 3rd line in Table 2. The particle diameter was 212 ± 31 nm after the additional steps including washing, ultrasonic sonication, and dilution steps to obtain a monodisperse sample. In the following, Fig. 1b approved the distribution and also the mean hydrodynamic particle size of the curcumin-niosomes nanoparticles. The corresponding particles showed the Z-average of 526 nm with the PDI number of 0.596. Also, a single peak with the diameter of 241 nm was appeared with the intensity of 57% representing the higher portion of the particles had a same size near 241 nm [57].

### Curcumin release profile from curcumin niosome nanoparticles

**Fig. 2:**
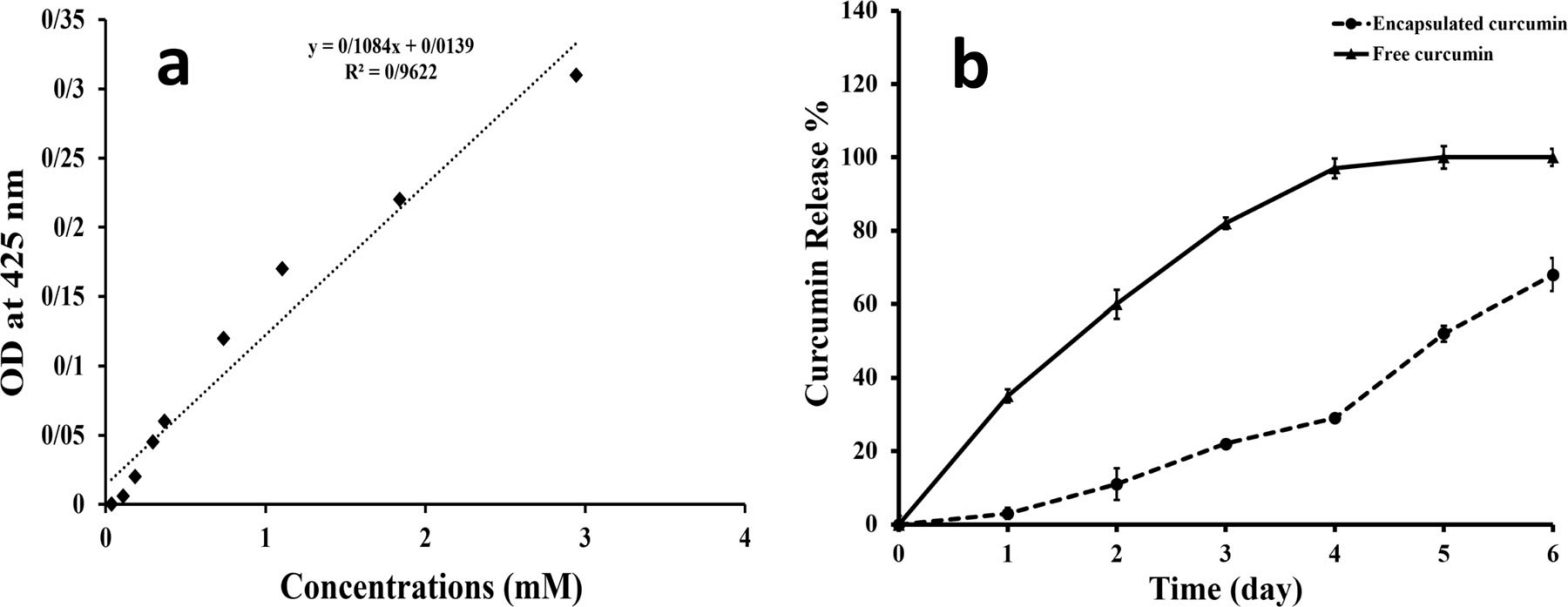
The standard curve related to the different concentrations of curcumin has a correlation coefficient of R2 as 0.96 guarantying the specificity of 425 nm for curcumin (**a**). The release pattern of the free and encapsulated curcumin indicates the lower slope for the encapsulated curcumin due to the controlled release of curcumin (**b**)

A standard curve was obtained using a concentration gradient from 0.036 to 3 mM (Fig. 2a). The samples with the predetermined curcumin concentrations were made and their absorbance at 425 nm was read. The resultant curve was employed for the analysis of curcumin release kinetic from the suspension of the niosome nanoparticles. The release was examined after 0, 1, 2, 3, 4, 5 and 6 days and illustrated as a curve in Fig. 2b. Table 3 summarized the correlation coefficients of the kinetic models. Also, in accordance with *R*^2^, the release mechanism of the encapsulated curcumin can be discussed. In contrast to the encapsulated group, the free curcumin indicated a linear diffusion rate that reached to a plateau of 100% after 4 days.

**Table 3:** The regression coefficients belonged to the different kinetic models of the curcumin release from the curcumin niosome nanoparticles

Correlation coefficients for release of curcumin from niosomes	Zero order	First order	Higuchi	Korsmeyer-Peppas
*R*^2^	0.91	0.76	0.99	0.95

### Stability of curcumin niosome nanoparticles

**Fig. 3:**
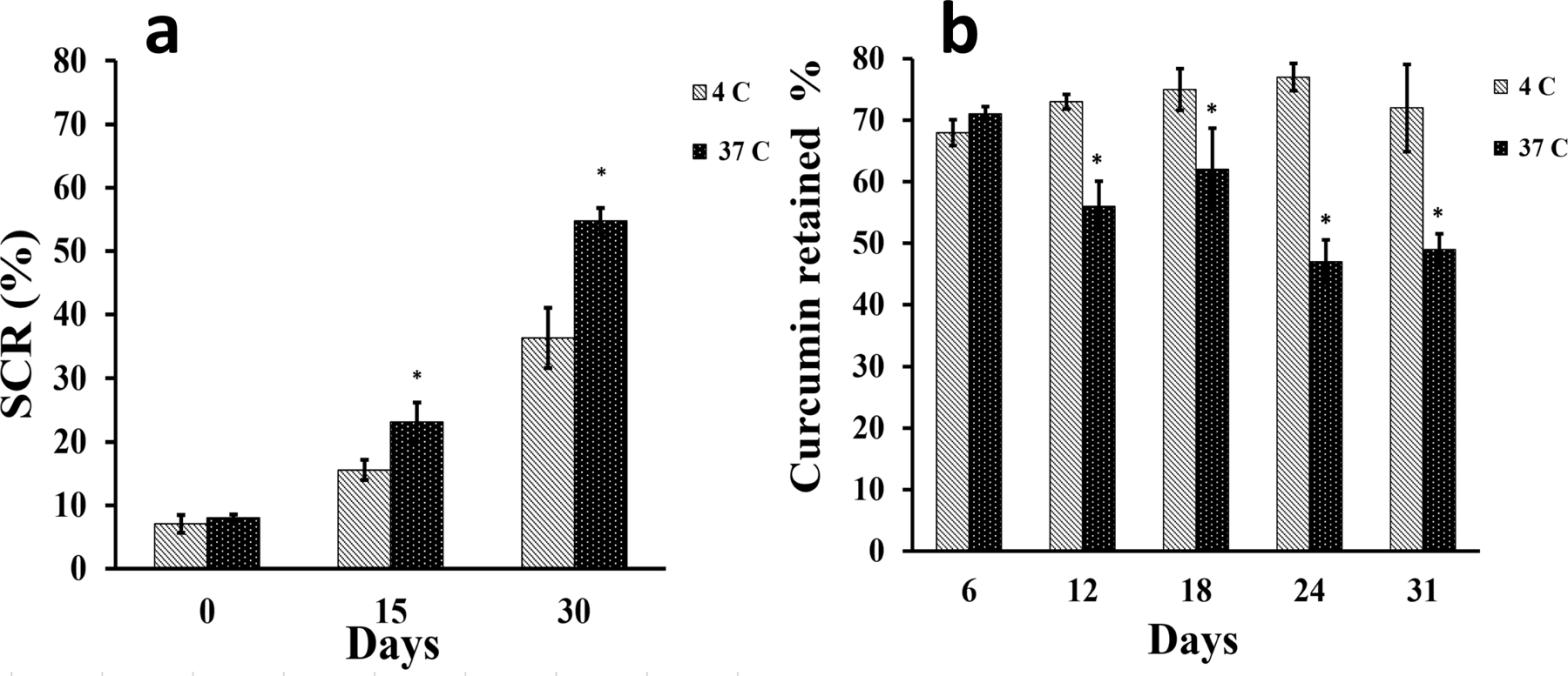
The stability of the curcumin niosome nanoparticles by the measurements of the particle size by SEM at 4 and 37 °C confirms that the lower diameter changes at 4 °C compared to 37 °C (**a**). The retained percent of curcumin (%) at 4 and 37 °C shows the lower degradation of curcumin as 68–77% when the sample is kept at the lower thermal condition (**b**)

The size of the curcumin-niosome nanoparticles increased after the incubation in 4 °C and 37 °C for 15 and 30 days (Fig. 3a). The precipitates started to be formed after 15 days at 37 °C and the fine pellet was detected at the 30th day of the incubation for the both incubation temperatures. However, by shaking, the pellet was disappeared easily, but after a while, it was created again. The relations between the two corresponding temperatures were significant at the all time points except after the first time point that the samples were incubated for 1 h. The data approved that if the samples were stored at a lower temperature, the storage stability of the prepared nanoparticles would be increased [58]. Moreover, the curcumin content of these nanoparticles were studied after 12, 18, 24 and 31 days at the both incubation temperature (Fig. 3b).

### Cell viability after the treatment with curcumin niosome particles

**Fig. 4:**
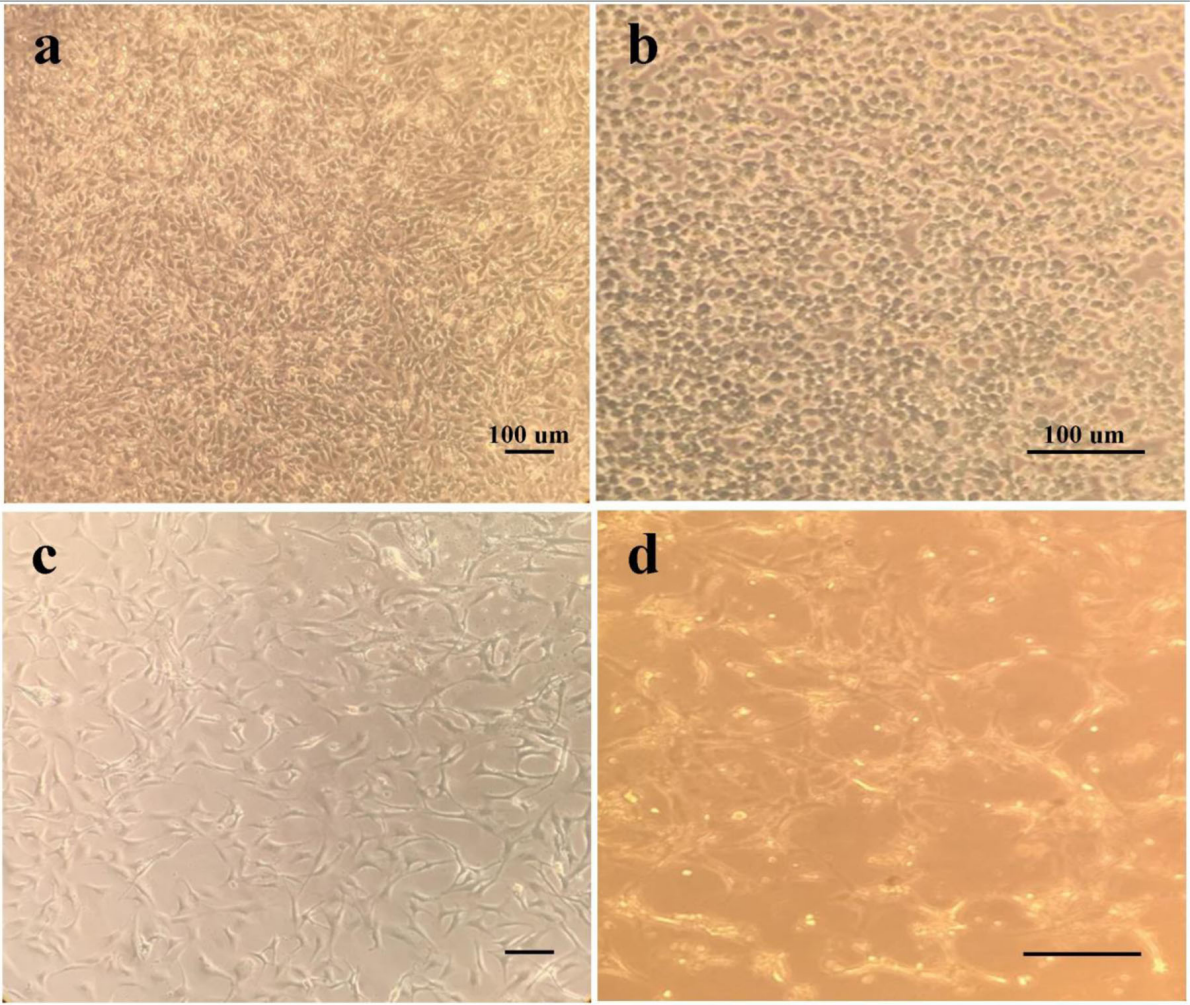
The cell morphology of TCPS (**a**), the cell morphology after the treatment with 2 µl (**b**), the cell morphology after the treatment with 7 µl (**c**) and the cell morphology after the treatment with 12 µl (**d**) of the batch curcumin niosome nanoparticles. Approximately, the cells are intact in (**a**–**b**) groups, while the cells of (**c**–**d**) confirm the apoptotic impact of the nanoparticle treatments. When, the groups of c and d are compared to each other, it results that the group which was treated by 7 µl, has cell death beside the enough cell preservation (50%) for Real-Time PCR. Thus, the corresponding dose was selected as IC50 point

**Fig. 5:**
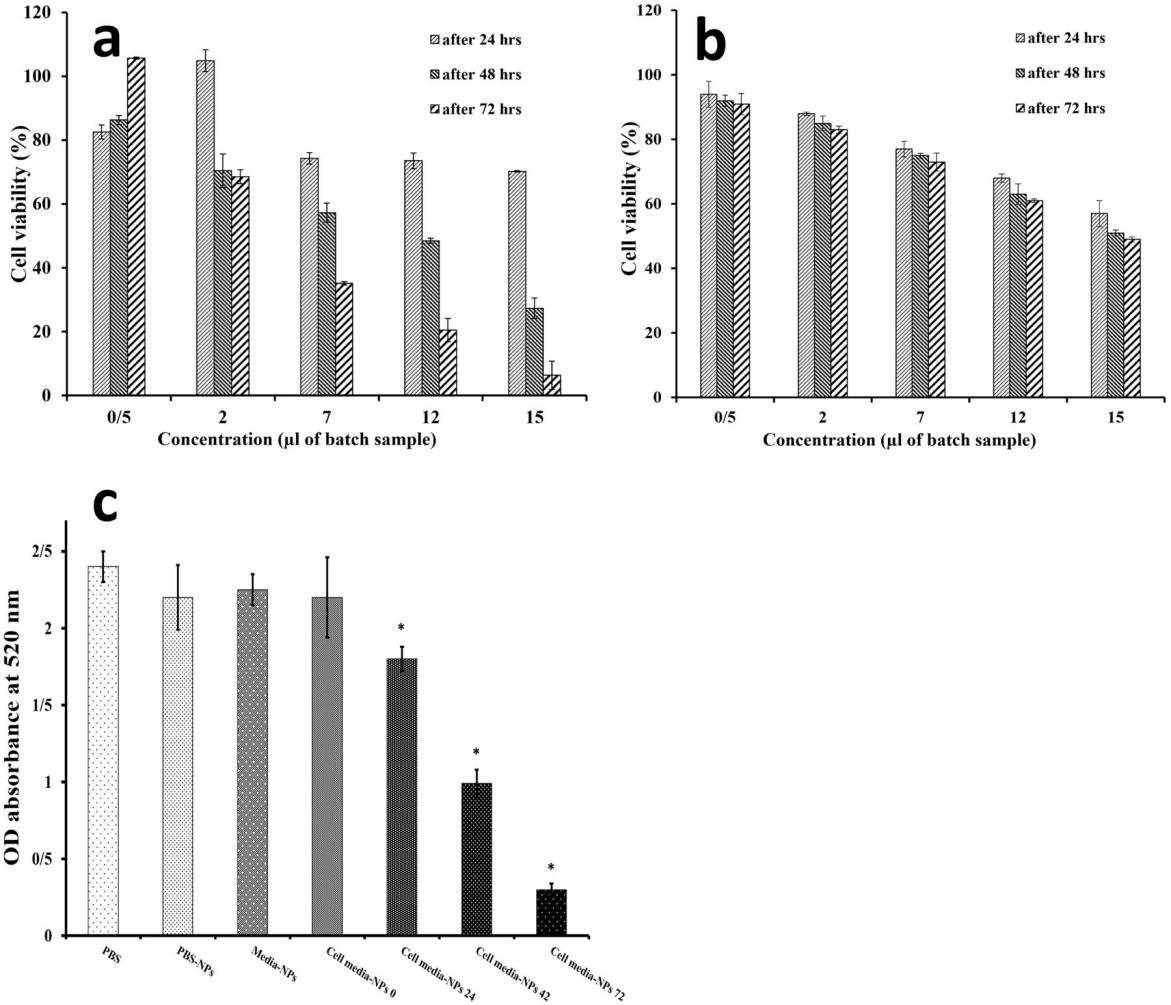
MTT analysis chart of the cells after the treatment with a serial dilution of the batch curcumin niosome nanoparticles on sw-1736 thyroid cancer cells (**a**) and normal Hu02 cell line (**b**). The IC50 points of the nanoparticles were 7 and 15 µl for sw-1736 thyroid cancer cells and normal Hu02 cell line respectively. DPPH assay before and after the addition of 7 µl of the batch curcumin niosome nanoparticles illustrates the presence of radicals due to the nanoparticle treatment. The star indicates the significant difference between the groups when *P* value is lower than 0.05 (**c**)

The morphological studies of sw-1736 thyroid cancer cell line were shown in Fig. 4. The cell groups were treated with 0, 0.5, 2, 7, 12 and 15 µl of the batch curcumin-niosome nanoparticles (2.28 mM) in 100 µl of cell culture media. The cell viability percepts were measured via MTT assay. The concentrations of 7, 12 and 15 µl showed the sharp morphological changes as shrinkage, irregular shape and the appearance of apoptotic bodies [59] after 72 h. The figure was related to 0, 2, 7 and 12 µl (Fig. 4a–d) of the curcumin-niosome nanoparticles. However, the cells which were treated with 15 µl, the whole cells were destroyed and removed during the washing step with PBS before MTT analysis. In accordance with the MTT results (Fig. 5a), every concentration made cell death to some extent. However, the concentrations of 7, 12 and 15 µl changed the normal cell shape to apoptotic morphology. The concentration of 2 µl led into cell death below than 50% of the seeded cells and the cell appearance was more similar to the control group (without any treatments). Moreover, the cells which were treated with 12 µl of the curcumin-niosome nanoparticles in 100 µl of cell culture media, made cell death more that 50% of the cultured cells, while the cell treatment group with 7 µl showed a mortality value of 50% and considered in this study as IC50 point [60]. Every cell death values of this group at the time points had significant relations compared to the control group (*p* value < 0.05). In other words, 7 µl of the batch curcumin-niosome sample in 100 µl of the cell culture media was enough for 50% growth inhibition of the thyroid cancer cell line and hence, 7 µl was selected for Real-Time PCR assay. On the other hand, the assay was repeated for Hu02 cell line as a normal cell line. The results approved the higher concentration of IC50 as 15 µl highlighting the lower sensitivity of these cells to the nanoparticles [61].

### Measurement of the created radical amount by DPPH assay

The radicals which was produced as a result of the cell treatment could be measured by DPPH assay. Herein, DPPH was dissolved in PBS, the PBS-nanoparticles, the cell culture media nanoparticles, the cell supernatant media-nanoparticles and their absorbance at 520 nm were read at 0 h. Only the absorbance of the cell supernatant media- nanoparticle group at 520 nm was studied after 24, 48 and 72 h (Fig. 5b) to quantify the produced radical value by the cells during time. The supernatant cell media was gathered from the treated cells at 4 time points including 0, 24, 48 and 72 h. This cell media could contain the free radicals, which were produced by the cells as a result of the released curcumin of the nanoparticles and these radicals may make the cellular apoptosis. The group contained only PBS, was control group for all experimental groups. The developed free radicals by the treated cells react with DPPH compound and accordingly, this reaction changes the purple color of DPPH to the yellow-colored diphenylpicryl hydrazine [62]. Thus, the absorbance value at 520 nm was depended on the free radical amount. In this manner, if the cells produce the higher amount of free radicals, the absorbance value will be smaller (Fig. 5b). Herein, only the absorbance values of the cell supernatant media showed a significant relation compared to the control group (DPPH + PBS) at the time points of 24, 48 and 72 h (*p* value < 0.05). However, the radicals of the curcumin-niosome nanoparticles were analyzed to determine the radical amount of curcumin itself in PBS. The cell culture media and the PBS-nanoparticles showed insignificant relations compared to the control group. Also, there was no a significant difference between the cell supernatant media-nanoparticles group and the control group at the time point of 0 h (*p* value ≈ 0.22).

### Analysis of apoptotic genes after the cell treatment with the curcumin niosome particles

**Fig. 6:**
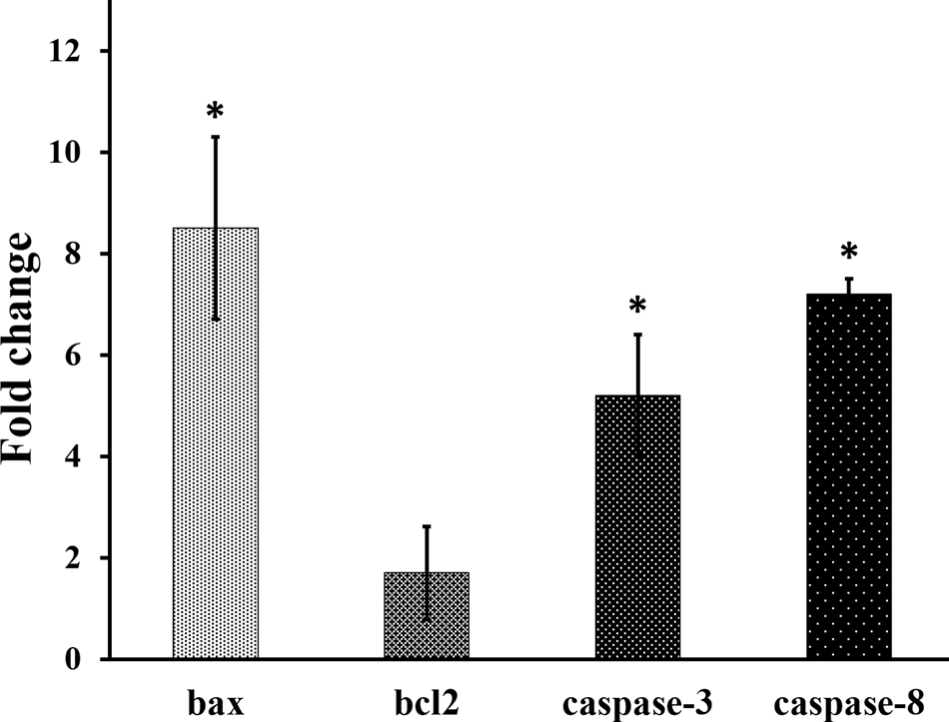
The gene expression of sw-1736 thyroid cancer cells after the treatment of 7 µl of the curcumin niosome nanoparticles for 72 h. The higher expression of bax, caspase-3 and caspase-8 in the treated group confirms the apoptotic impact of the nanoparticles. Moreover, the fold change of bcl2 as an inhibitor of cell death near to 1 shows the non-activation of this gene by the nanoparticles. The star indicates the significant difference between the groups (*P* value < 0.05)

Herein, the expression of bax, bcl2, caspase-3 and caspase-8 genes of sw-1736 thyroid cancer cell line was examined after the incubation with 7 µl of the batch curcumin-niosome nanoparticles in 100 µl of cell culture media for 72 h (Fig. 6). The all gene expression was calibrated with beta2M as the internal control gene. Herein, the expression of these genes in the experimental group which was treated with 7 µl of the curcumin-niosome nanoparticles per 100 µl of cell culture media was normalized with the non-treated control group. Figure 5 shows the over expression of bax as the value of 8.2 times compared to the control group (*p* value < 0.05) confirming the apoptotic destination of the corresponding cell line. However, bcl2 as an inhibitor of cell death had fold change ≈ 1.2 against the control group but not statistically significant (*p* value ≈ 0.35). Some studies suggested to calculate the ratio of bax/bcl2 [63] equals to 6.83 confirming apoptotic mechanism for cell death after the treatment with the curcumin-niosome particles. Accordingly, the genes inclusive caspase-3 and caspase-8 expressed in the test group as 4.8 and 6.9 respectively that the both values were higher in this group compared to the control group (*p* value < 0.05).

## Discussions

In accordance with before researches, niosomes with the diameter of 100–3000 nm are classified as large unilamellar vesicles (LUV) [64]. Herein, the prepared nanoparticles showed 241 and 212 nm by using DLS and SEM techniques respectively. Thus, these nanoparticles could be called as LUV. Also, when the PDI value of DLS method will be lower than 0.7, the dispersion of nanoparticles is monodisperse [65]. In contrast, the value of higher than 0.7 represents a broad distribution [66]. The corresponding value of the prepared nanoparticles was 0.596 confirming a monodisperse dispersion of nanoparticles. In other words, the PDI value of lower than 0.7 indicates that DLS is reliable and guarantees that the sample is monodisperse [67]. About the curcumin encapsulation efficiency, there are many factors may change the curcumin entrapment. The corresponding factors could be listed as component types, their molecular weight and also the concentrations of these components. As a result, the addition of cholesterol made the development of the noisomes as well as the higher entrapment efficiency of curcumin [68]. Moreover, cholesterol could enhance the stability of the prepared niosomes compared to the synthesis without cholesterol. On the other hand, PEG6000 as a function of its higher molecular mass compared to PEG600, increased the stability of the niosomes [69]. It has been reported that curcumin in water forms hydrogen bonds by its side groups [70]. On the other hand, PEG is commonly used for the curcumin encapsulation as a co-solvent [71]. The mechanism of the higher curcumin solubility in the presence of PEG may be occurred by PEG attachment to the hydrophilic part of curcumin and partitioning them in the solution [72]. In other words, the trapping of curcumin into PEG, increases its solubility even about 2000 fold compared to curcumin without PEG in water [73] or ethanol. The similar interaction between curcumin and other molecules as starch [74] was reported frequently. Another property of PEG for the curcumin encapsulation is the higher stability of the nanoparticles due to its steric stabilization and also, its ability to attach the membrane components as Tween and Span [75]. It is clear that these events could increase the curcumin encapsulation after the addition of PEG. The results of the encapsulation efficacies of the formulations approved that PEG and cholesterol have a synergic effect and they could enhance the curcumin entrapment value to 76%. Also, the loading capacity of the nanoparticles was the maximum value when the nanoparticles were developed in the presence of both PEG and cholesterol (Table 2). This loading capacity indicated that only 16.8% of the nanoparticles is loaded by curcumin and also, within 1 mg of the nanoparticles, there is 0.0168 mg of curcumin. Previous studies reported that the release of curcumin from niosome particles is strictly depended on the ratio of hydrophobic components such as Tween-100, span 80 and cholesterol to curcumin [76]. On the other hand, due to the hydrophobic nature of curcumin, it was expected that its release percent would be controlled in water. The hydrophobic nature of curcumin beside its entrapment within niosomes, the diffusion of this compound would be limited [77]. In addition, it was reported that the presence of cholesterol can reduce drug leaky due to its filling effect of niosome pores [76]. In this assessment, Peppas equation was obtained as *Y* = 11 X − 19; *R*^2^ = 0.95 with *n* = 1 approving super case II transport mechanism for the curcumin release. This phenomena is related to the limiting role of the curcumin release from the niosome particles [78]. The result was confirmed again when the correlation value of higuchi model was obtained as 0.99 [79]. Higuchi model describes the release mechanism through fickian diffusion [80]. In this relation, the release rate is controlled in accordance with the square root of time. In addition, the curcumin release follows the zero order kinetic law which is resulted when a formulation employs Tween-80 and cholesterol simultaneously [76]. After 6 days, about 68% of the encapsulated curcumin in the niosomes particles, was released confirming a sustained release model of the drug. However, the free curcumin reached to a plateau after only 4 days that explains the fast release mechanism in this group. About the nanoparticle stability, it has been reported when the SCR value is lower than 10%, the prepared particles will be stable [81]. In this study, the particle stability reduced after the 15th day of the incubation at the both incubation temperatures. It could be suggested that for the higher stability of nanoparticles, niosome particles should be lyophilized and stored at 4 °C [82]. In this manner, the curcumin amount of the samples which were kept at 4 °C, was higher than the samples at 37 °C. The curcumin percentage which was retained at 4 °C, was between 68 and 77%, while the value would be decreased to 47% when the particles were incubated at 37 °C [83]. As a whole, the data showed that the nanoparticles stability decreased during the time and the temperature had a significant effect. For the development of the nanoparticles for cancer therapy aim, it is not enough that the optimized nanoparticles have a uniform size with an appropriate encapsulation efficiency. Thus, they should be effective for cell death and change cell shape to apoptotic morphology. In accordance with before studies, the morphological criteria including apoptotic cell rounding, cell shrinkage [84] and the rupture of plasma membrane with the liberation of cytoplasmic content [85] confirm cell apoptosis. These morphological alterations could be detected using a light microscopy [86, 87] or SEM [88] or transmission electron microscopy (TEM) [89]. 4′ 6‐diamidino‐2‐phenylindole (DAPI) staining specifically stains cell nucleus, whereas cell morphological studies examine cells entirely to detect apoptosis changes [90]. Even, if we are interested on nucleus alterations during cell apoptosis, DAPI staining could not label the fragmented portions of DNA which are created in the later stages of apoptosis [91]. Herein, the IC50 concentrations were 7 and 15 µl for the cancer and normal cell lines. The mechanism of lower toxicity against normal cell lines compared to cancer cell lines had been approved by most studies [61, 92–94]. Some groups reported that the corresponding difference has been related to the higher sensitivity of cancer cells to nanoparticles [61] due to their higher metabolism. Also, the higher absorption of nanoparticles by cancer cells could be another reason. The mechanism that makes a higher nanoparticle concentration is needed for the death of normal cells, may depend on their different gene expression about membrane proteins [95]. These proteins are responsible for the uptake of nanoparticles. However, the mechanism has not been discussed exactly. The measurement of the radicals which were produced by sw-1736 cell line after the treatment, could help to find out death mechanism. Thus, the radicals were measured by DPPH assay in this study and the results indicated that the amount of the generated radicals was increased during the time. These radicals could make alterations in DNA sequence and finally leads to apoptosis [96]. In particular, the gene expression of the treated thyroid cell line indicated the expression profile of apoptosis (Fig. 6). Among the examined genes in this study, bcl2 inhibits cell death rather than cell proliferation and bax is linked to cell apoptosis induction [97]. Thus, the lower ratio of this gene leads into the resistance against anticancer condition [98]. In addition, due to the activation of caspase-3 by bcl2 family, it was known as an apoptotic marker [99]. Also, caspase-8 as an initiator of caspase-3 could increase cell apoptosis and the up-regulation of this gene augments cell death [100]. However, some studies confirmed the activation of caspase-3 by caspase-8 [101]. Finally, in the treated cells, the expression of the apoptotic markers including bax, caspase-8 and caspase-3 was higher compared to the non-treated cells. Finally, all physicochemical properties of the optimized niosome nanoparticles were gathered in Table 4.

**Table 4:** All physicochemical properties of the curcumin niosome nanoparticles

Encapsulation efficiency (%)	Loading capacity (%)	Released value (%)	DLS PDI	DLS diameter (nm)	SEM diameter (nm)	IC50 (mM)	Minimum DPPH value (OD)	Bax /bcl2 ratio
76 ± 14.5	16.7 ± 1.6	68 ± 4.5	0.5 ± 0.15	241 ± 13	212 ± 31	0.159	0.3 ± 0.04	6.83 ± 1.99

## Conclusions

In spite of the increasing rate of thyroid cancer incidence, the number of studies in this field is not satisfying. Moreover, drug based therapeutic methods are not available to combat thyroid cancers and a few studies have been established based on curcumin effects around thyroid anomalies. Herein, the formulation of the curcumin nanoparticles was optimized to get a monodisperse distribution. The nanoparticles showed a controlled release of curcumin after the formulation had been modified with PEG6000. It is worth to note that there was a synergic manner between PEG and cholesterol to obtain this controlled release pattern of curcumin. If PEG is removed from the formulation, the curcumin release will be inhibited drastically. Also, PEG decreases the activation of immune system when the particles will be used intravenously. The curcumin release followed fickian diffusion and also, super case II transport. These mechanisms define that the curcumin release is related to the curcumin concentrations in the media and also, the erosion of the nanoparticles respectively. It should be noted that when PEG as a degradable polymer is employed for the formulation of the nanoparticles, its destruction will induce the curcumin release. The stability of the optimized formulation was higher at 4 °C compared to 37 °C. However, the incubation time at the both temperature conditions indicates that the nanoparticles will have enough time to influence on cell fate before their decomposition. Also, the corresponding nanoparticles were effective on cell death and increased the amount of cell radicals. Moreover, the gene expressions of the treated cells were near to apoptotic fate and DPPH assay presented the production of radicals by the treated cells. Finally, the study could open a new way for the therapeutic aim of thyroid cancer that has attracted a few studies unfortunately. It is clear that this study needs other studies covering at least protein signaling and in vivo studies.
